# Supplementary material for: Potential geographic distribution of relict plant Pteroceltis tatarinowii in China under climate change scenarios
Source: PLoS One. 2022 Apr 8;17(4):e0266133. doi: 10.1371/journal.pone.0266133 (PMC8993005; doi:10.1371/journal.pone.0266133)
Supplement: S1 Table — (DOCX) [file pone.0266133.s002.docx]

**S2 Table.** Percent contribution of 19 bioclimatic variables

| Variable | Percent contribution (%) |
| --- | --- |
| bio13 | 42.57 |
| bio6 | 30.13 |
| bio3 | 9.43 |
| bio15 | 3.52 |
| bio4 | 3.18 |
| bio7 | 3.15 |
| bio19 | 3.05 |
| bio10 | 0.95 |
| bio11 | 0.88 |
| bio18 | 0.55 |
| bio1 | 0.49 |
| bio2 | 0.49 |
| bio14 | 0.48 |
| bio17 | 0.37 |
| bio9 | 0.29 |
| bio5 | 0.23 |
| bio16 | 0.12 |
| bio8 | 0.08 |
| bio12 | 0.03 |
