# Supplementary material for: Potential geographic distribution of relict plant Pteroceltis tatarinowii in China under climate change scenarios
Source: PLoS One. 2022 Apr 8;17(4):e0266133. doi: 10.1371/journal.pone.0266133 (PMC8993005; doi:10.1371/journal.pone.0266133)
Supplement: S2 Table — (DOCX) [file pone.0266133.s003.docx]

**S3 Table. Pairwise Pearson’s correlation coefficients of environmental variables.**

|  | **bio1** | **bio2** | **bio3** | **bio4** | **bio5** | **bio6** | **bio7** | **bio8** | **bio9** | **bio10** | **bio11** | **bio12** | **bio13** | **bio14** | **bio15** | **bio16** | **bio17** | **bio18** |
| --- | --- | --- | --- | --- | --- | --- | --- | --- | --- | --- | --- | --- | --- | --- | --- | --- | --- | --- |
| **bio2** | -0.565 |  |  |  |  |  |  |  |  |  |  |  |  |  |  |  |  |  |
| **bio3** | -0.024 | 0.431 |  |  |  |  |  |  |  |  |  |  |  |  |  |  |  |  |
| **bio4** | -0.55 | 0.684 | -.273 |  |  |  |  |  |  |  |  |  |  |  |  |  |  |  |
| **bio5** | 0.596 | 0.114 | -0.07 | 0.232 |  |  |  |  |  |  |  |  |  |  |  |  |  |  |
| **bio6** | 0.911 | -0.762 | 0.001 | -0.701 | 0.275 |  |  |  |  |  |  |  |  |  |  |  |  |  |
| **bio7** | -0.603 | 0.792 | -0.124 | 0.976 | 0.197 | -0.851 |  |  |  |  |  |  |  |  |  |  |  |  |
| **bio8** | 0.285 | 0.225 | 0.159 | 0.173 | 0.534 | 0.061 | 0.16 |  |  |  |  |  |  |  |  |  |  |  |
| **bio9** | 0.948 | -0.696 | -0.006 | -0.722 | 0.404 | 0.973 | -0.769 | 0.075 |  |  |  |  |  |  |  |  |  |  |
| **bio10** | 0.791 | -0.164 | -0.152 | -0.013 | 0.926 | 0.525 | -0.069 | 0.807 | 0.631 |  |  |  |  |  |  |  |  |  |
| **bio11** | 0.942 | -0.701 | 0.031 | -0.763 | 0.349 | 0.983 | -0.805 | 0.117 | 0.982 | 0.59 |  |  |  |  |  |  |  |  |
| **bio12** | 0.782 | -0.726 | -0.187 | -0.65 | 0.291 | 0.84 | -0.702 | -0.169 | 0.873 | 0.499 | 0.822 |  |  |  |  |  |  |  |
| **bio13** | 0.715 | -0.593 | -0.148 | -0.533 | 0.33 | 0.751 | -0.6 | 0.006 | 0.768 | 0.485 | 0.734 | 0.871 |  |  |  |  |  |  |
| **bio14** | 0.760 | -0.685 | -0.357 | -0.474 | 0.393 | 0.761 | -0.542 | -0.165 | 0.812 | 0.586 | 0.748 | 0.938 | 0.761 |  |  |  |  |  |
| **bio15** | -.503 | .555 | 0.51 | 0.226 | -0.3 | -0.473 | 0.278 | 0.253 | -0.534 | -0.433 | -0.471 | -0.695 | -0.4 | -0.82 |  |  |  |  |
| **bio16** | 0.781 | -0.710 | -0.097 | -0.704 | 0.25 | 0.864 | -0.755 | -0.063 | 0.869 | 0.451 | 0.842 | 0.944 | 0.954 | 0.827 | -0.495 |  |  |  |
| **bio17** | .751 | -.664 | -.361 | -0.446 | 0.41 | 0.74 | -0.512 | -0.171 | 0.799 | 0.592 | 0.729 | 0.932 | 0.749 | 0.992 | -0.848 | 0.815 |  |  |
| **bio18** | 0.672 | -0.667 | -0.065 | -0.692 | 0.14 | 0.777 | -0.741 | 0.0759 | 0.754 | 0.339 | 0.75 | 0.824 | 0.898 | 0.66 | -0.353 | 0.922 | 0.647 |  |
| **bio19** | 0.762 | -0.661 | -0.318 | -0.476 | 0.408 | 0.758 | -0.537 | -0.148 | 0.814 | 0.592 | 0.746 | 0.942 | 0.761 | 0.991 | -0.728 | 0.828 | 0.992 | 0.663 |

**Notes:**

bio1: Annual Mean Temperature; bio2: Mean Diurnal Range; bio3: Isothermality; bio4: Temperature Seasonality; bio5: Max Temperature of Warmest Month; bio6: Min Temperature of Coldest Month; bio7: Temperature Annual Range; bio8: Mean Temperature of Wettest Quarter; bio9: Mean Temperature of Driest Quarter; bio10: Mean Temperature of Warmest Quarter; bio11: Mean Temperature of Coldest Quarter; bio12: Annual Precipitation; bio13: Precipitation of Wettest Month; bio14: Precipitation of Driest Month; bio15: Precipitation Seasonality; bio16: Precipitation of Wettest Quarter; bio17: Precipitation of Driest Quarter; bio18: Precipitation of Warmest Quarter; bio19: Precipitation of Coldest Quarter.
