# Supplementary material for: Potential geographic distribution of relict plant Pteroceltis tatarinowii in China under climate change scenarios
Source: PLoS One. 2022 Apr 8;17(4):e0266133. doi: 10.1371/journal.pone.0266133 (PMC8993005; doi:10.1371/journal.pone.0266133)
Supplement: Attachment — Submitted filename: Response to reviewers.docx [file pone.0266133.s004.docx]

Dear Editors and Reviewers:

We would like to thank the editor for giving us a chance to resubmit our manuscript entitled “Prediction of Potential Geographic Distribution of Relict Plant *Pteroceltis tatarinowii* in China Based on MaxEnt” (ID: PONE-D-21-32985), and also thank the reviewers for giving us constructive suggestions which would help us both in English and in depth to improve the quality of the paper. Here we submit a new version of our manuscript with the title “Potential Geographic Distribution of Relict Plant *Pteroceltis tatarinowii* in China under Climate Change Scenarios”, which has been modified according to the reviewers’ suggestions. Efforts were also made to correct the mistakes and improve the English of the manuscript. We mark all the changes in red in the revised manuscript. The main corrections in the paper and the responds to the reviewer’s comments are as flowing:

**Responds to the comments of Reviewer #2:**

1. **Comment**: Abstract seems to qualitatively stated. Pls speak numerically.

**Response**: Thank you for your valuable advice. The abstract has been modified and stated numerically. **(L35-L39)**.

2. **Comment:** (The authors predicted the future using the emission scenarios. How about the past? What happened during the calibration period?)

**Response:** Thank you for your valuable advice. In the discussion, we pointed out the significance of simulating past scenarios and the limitation of this study. (**L405-L412**).

.

3. **Comment:** The title says site suitability analysis for Pteroceltis tatarinowii. Should the paper only be concerned with this? The first sentence of the introduction gives me the impression that authors are evaluating the impact of climate change on the distribution of this plant. If so, the title and abstract need to change accordingly.

**Response:** Thank you for your valuable advice. We revised the title to “Potential Geographic Distribution of Relict Plant *Pteroceltis tatarinowii* in China under Climate Change Scenarios” .

4. **Comment:** The authors need to provide a better literature review on Maxent application in china the results obtained. The advantages and disadvantages of this model should be provided in comparison with other similar models.

**Response:** Thank you for careful work. We provide a better literature review in Introduction (**L72-L81**).

5. **Comment:** I believe the intro part for the Pteroceltis tatarinowii species could be briefed..

**Response:** Thank you for your careful work. As Reviewer suggested that the intro part for the *Pteroceltis tatarinowii* species was briefed (**L82-L91**).

6. **Comment:** What is the innovation of this research?.

**Response:** As Reviewer suggested that the innovation of this research was supplemented in discussion part (**L420-L442**).

**7. Comment:** The methods section could be improved. The readers are only concerned with the general flow of the work. Small details like saving a file in .CSV format is not relevant.

**Response:** Thank you for your careful work. The methods section was improved according to reviewer’s comment **(L127, L138-L139, L146, L155)**

8. **Comment**: How did you identify the inaccurate sites in the distribution records of P. tatarinowii. What criteria did you use?

**Response**: The method to identify the inaccurate site was added in **L120-L127**

9. **Comment:** Figure 1 legend if not informative. What are those high and low values?

**Response**: Thank you for your careful work. The information in the legend of Figure 1 has been improved (**Fig. 2**)

10. If possible, use a graded symbology for the distribution points in terms of frequency. Larger circles indicating higher numbers of this plant. This way the reader can guess which part of China are more suitable for this plant.

**Response:** As Reviewer suggested that a graded symbology for the distribution points in terms of frequency. (**Fig. 2**)

.

10. **Comment:** If the study area is the whole country of china, I believe the first part of the methods section must be concerned with describing the distribution of environmental factors across China.

**Response:** Thank you for your valuable advice. As Reviewer suggested that the describing of climate factors across China was added in **L105-L116**.

11. **Comment:** “…scenarios could describe the prediction results of future climate change more scientifically.” Citation pls.

**Response:** Thank you for your careful work. Citation was supplemented (**L139**).

12. **Comment:** The terms used in table 1 should be clarified in the text.

**Response:** Thank you for your careful work. We have clarified.the terms used in Table 1.

13. **Comment:** It would be useful to have a flowchart for the analysis. The details can be provided in this chart instead of the text..

**Response:** .Thank you for your valuable advice. We have supplemented a flowchart. (**Fig. 1**)

14. **Comment:** “… The closer the AUC ...simulation accuracy were divided into four grades: poor….” Citation pls.

**Response:** Thank you for your careful work. Citation was added. (**L168, L170**)

15. **Comment:** Section 3.2 is the repetition of Fig 6. Pls provide only the results not noticeable on the map.

**Response:** We have revised the expression of section 3.2 **(L248-L249, L251-L253).**

16. **Comment:** Based on your results in table 2, precipitation in China will increase significantly in the future under the climate change. The results in this table are only valid if there will be no more expansion of human activities in China which is highly unlikely.

**Response:** As Reviewer suggested that the influence of human activities was discussed in section 4.3 (**L412-L419)**.

17. **Comment:** A lot of the results are repeated in the discussion section. Pls only elaborate on your findings and avoid restating the results. This section has become long and tedious.

**Response:** Thank you for your careful work. We revised the discussion part and deleted the repeated part.

18. **Comment:** English usage needs serious improvement.

**Response:** Thank you for your careful work. We have made every effort to improve the use of English.

We tried our best to improve the manuscript and made some changes in the manuscript. These changes will not influence the content and framework of the paper. And here we did not list the changes but marked in red in revised paper.

We appreciate for Editors/Reviewers’ warm work earnestly, and hope that the correction will meet with approval.

Once again, thank you very much for your comments and suggestions.
