# Supplementary material for: Potential geographic distribution of relict plant Pteroceltis tatarinowii in China under climate change scenarios
Source: PLoS One. 2022 Apr 8;17(4):e0266133. doi: 10.1371/journal.pone.0266133 (PMC8993005; doi:10.1371/journal.pone.0266133)
Supplement: Attachment — Submitted filename: Response to reviewers.docx [file pone.0266133.s005.docx]

Dear Editors and Reviewers:

We would like to thank the editor for giving us a chance to resubmit our manuscript entitled “Prediction of Potential Geographic Distribution of Relict Plant *Pteroceltis tatarinowii* in China Based on MaxEnt” (ID: PONE-D-21-32985), and also thank the reviewers for giving us constructive suggestions which would help us both in English and in depth to improve the quality of the paper. Here we submit a new version of our manuscript with the title “Potential Geographic Distribution of Relict Plant *Pteroceltis tatarinowii* in China under Climate Change Scenarios”, which has been modified according to the reviewers’ suggestions. Efforts were also made to correct the mistakes and improve the English of the manuscript. We mark all the changes in red in the revised manuscript. The main corrections in the paper and the responds to the reviewer’s comments are as flowing:

**Responds to the comments of Reviewer #4:**

1. **Comment**: Page4, line 127: “second” this part might be needs a reference to confirm your method.

**Response**: Thank you for your valuable advice. A reference has been added to confirm my method **(L127)**.

2. **Comment:** Please explain why choice environment variables from worldclim?

**Response:** Thank you for your valuable advice. We have explained the reasons for choosing to download variables from worldclim and supplemented the corresponding references. (**L132-L134)**.

3. **Comment:** Where you get the Al, aspect and slope?

**Response:** The sources of altitude, slope and aspect data have been supplemented. (**L135-L136)**.

.

4. **Comment:** page4, section 2.3: Considering the uncertainty of future climate scenarios, assessments of the impact should incorporate data from a range of climate models that represent different climate sensitivities to various possible future climate change projections. Thus, to reduce this impact on global circulation models (GCMs) on which the future climate datasets are based, an ensemble forecasting procedure was need to use in current study.

**Response:** Thank you for your valuable advice. In **L139-L140**, the reasons for choosing BCC-CSM2-MR model have been supplemented..

5. **Comment:** page5, line144：please list your manuscript. How to implement the Spearman correlation analysis? Which software? Please list in manuscript.

**Response:** We provide the result of Spearman correlation analysis in **Table S1**.

6. **Comment:** page5, section 2.4: please list the detail parameters of maxent were used in your study.

**Response:** Thank you for your careful work. As Reviewer suggested that the detail parameters of maxent was supplemented (**L157-L162**).

7. **Comment:** page5, section 2.5: AUC have many disadvantages to assess the performance of model, especially for Maxent. Thus, the pROC, TSS or Kappa were used to assess the model.

**Response:** As reviewer suggested that the we have explained this point in the discussion and added supporting literature. (**L349-L357**).

**Responds to the comments of Reviewer #5:**

**1. Comment:** I imagine that “absence points” (together with those of occurrence) were also used to build the model but nothing is said about this in the text. Please also clarify this aspect which is not mentioned in the text (how they were generated or directly detected).

**Response:** An important feature of MaxEnt model is that only ‘no-absence points’ is required for modeling, which is supplemented in the introduction..**(L75-L76)**

2. **Comment**: even the creation of the buffer does not convince me since it is a relict tertiary plant. why was it necessary to do this (and how does it relate to the altitude, slope and aspect reported in table 1?

**Response**: Thank you for your valuable advice. The method for selecting distribution points was provided in **L123-L127.**

3. **Comment:** all variables were imported into the MaxEnt and repeated 3 times (L141): pls justify this procedure.

**Response**: Thank you for your careful work. We confirmed this procedure.

\

8. 4. **Comment:** In the method section “223 effective distribution points of P. tatarinowii and 11 environmental variables” were considered, but in the results only “96 distribution points” were mentioned. What happened?

**Response:** Thank you for your careful work. It should be 223 points (**L127, L193**)

.

5. **Comment:** L322: the authors refer of "the gravity center of P. tatarinowii's potential suitable habitat"; this treatment upsets me and I do not understand how it is possible to associate the potential habitat of a tertiary relict plant with a “center of gravity”. They seem to me to be two extremely different and unrelated concepts. My suspicion is that a good graphic picture of the model is being used to try to define something ecologically much more complex. So that, the authors refer to “potential geographical distribution (L388). The authors should clarify this fundamental aspect. Curiously, in fact, the authors declare (LL363-366) that “vegetation coverage, also have certain influence on the potential geographical distribution of P. tatarinowii. However, this variable is not included in the simulation because ... .. "but this is a primary trait that defines the habitat of the species. There is a lot of literature on this aspect which should be carefully considered.

**Response:** Thank you for your valuable advice. As Reviewer suggested that we have supplemented the research methods and references of gravity center in the method part.(**L187-L189)**.

6. **Comment:** the authors speak of a rare species but I observe very large distribution areas and therefore we should speak only of threatened species (specifying what it is threatened by).
.

**Response:** Thank you for your careful work. After our verification, *P. tatarinowii* is a unique relic plant in China.

7. **Comment:** L54-57: this sentence is not clear and, indeed, in its present form it seems just wrong / contradictory.

**Response:** We have rewritten this sentence (**L54-L55**)

8. **Comment:** L58; pls add the year after IPCC

**Response:** We have added the year after IPCC(L57)

9. **Comment:** L72-73: …among others

**Response:** As reviewer suggested that we have corrected the sentence (**L72**)

10. **Comment:** - L82: add patronymic

**Response:** We have add patronymic **(L81).**

11. **Comment:** -L86: I believe that the ecological interest prevails over the other two.

**Response:** As Reviewer suggested that we have rewritten this sentence (**L85)**.

12. **Comment:** L89: only one population?

**Response:** Thank you for your careful work. We have corrected it (**L88**).

13. **Comment:** L99-101: delete this sentence

**Response:** Thank you for your valuable advice. We have deleted this part.

14. **Comment:** L118: the occurrences of

**Response:** We have corrected it according to reviewer’s advice (**L116**)

15. **Comment:L373:** add year of publication

**Response:** We have added year of publication in **L393**.

16. **Comment:** L391: meta-analysis

**Response:** We have corrected in **L410**.

17. **Comment:** L405: not all tertiary relict plants are “living fossil plants”.

**Response:** Thank you for your careful work. We have corrected in **L424**.

18. **Comment:** L422: mini?.

**Response:** Thank you for your careful work. We have corrected it in **L440**.

19. **Comment:** L418: one of the main results of this study is that “the future suitable area of P. tatarinowii simulated in this study is wider than the actual situation”. this seems counterintuitive to me

**Response:** We explained this conclusion in the discussion (L435-L437).

We tried our best to improve the manuscript and made some changes in the manuscript. These changes will not influence the content and framework of the paper. And here we did not list the changes but marked in red in revised paper.

We appreciate for Editors/Reviewers’ warm work earnestly, and hope that the correction will meet with approval.

Once again, thank you very much for your comments and suggestions.
